# *Drosophila* exercise, an emerging model bridging the fields of exercise and aging in human

**DOI:** 10.3389/fcell.2022.966531

**Published:** 2022-09-09

**Authors:** Meng Ding, Hongyu Li, Lan Zheng

**Affiliations:** Key Laboratory of Physical Fitness and Exercise Rehabilitation of Hunan Province, Hunan Normal University, Changsha, China

**Keywords:** *Drosophila*, exercise, aging, obesity, cardiac aging, lipid metabolism

## Abstract

Exercise is one of the most effective treatments for the diseases of aging. In recent years, a growing number of researchers have used *Drosophila* melanogaster to study the broad benefits of regular exercise in aging individuals. With the widespread use of *Drosophila* exercise models and the upgrading of the *Drosophila* exercise apparatus, we should carefully examine the differential contribution of regular exercise in the aging process to facilitate more detailed quantitative measurements and assessment of the exercise phenotype. In this paper, we review some of the resources available for *Drosophila* exercise models. The focus is on the impact of regular exercise or exercise adaptation in the aging process in *Drosophila* and highlights the great potential and current challenges faced by this model in the field of anti-aging research.

## 1 Introduction

Exercise is a serious challenge to systemic homeostasis and it causes a wide range of effects in a variety of cells, tissues and organs (Hawley et al., 2014). Indeed, a single exercise session is sufficient to produce acute changes at the transcriptional level (Williams et al., 1996), (Pilegaard et al., 2003). Multiple repetitions of exercise can produce exercise adaptation and more lasting effects on protein function (McGee and Hargreaves, 2020). Planned regular exercise can delay the development of chronic metabolic diseases, including cardiovascular diseases (CVDs), type 2 diabetes (T2D), insulin resistance and obesity (Hawley and Krook, 2016), (Fiuza-Luces et al., 2018), (Umpierre et al., 2011), (Goodyear and Kahn, 1998), (Castaño et al., 2020), (Houghton et al., 2017).

Aging is a major risk factor for CVDs, T2D and neurodegenerative diseases (Paneni et al., 2017), (Laiteerapong et al., 2019), (Hou et al., 2019). Epidemiological studies have shown that the increase in human lifespan has led to a high incidence of aging-related diseases, which places a huge burden on the world health care system (Partridge et al., 2018). Therefore, healthy aging will become one of the most important goals to be addressed today. Aging is determined by complex interactions between biology, environment, and society, which are beyond the control of the individual (Myint and Welch, 2012). But, lifestyle interventions can help to maintain health, such as increasing exercise as well as controlling diet (Partridge et al., 2018). It is well known that lifespan has heritable properties and therefore has a genetic basis. This was shown in studies in *Drosophila*, where differences in lifespan can be almost twofold across genetic backgrounds and these differences are heritable, supporting the model of genetic determination of lifespan (Campisi et al., 2019). *Drosophila* has powerful genetic tools and short lifespan characteristics, which make it an ideal model organism for studying lifespan and aging (Helfand and Rogina, 2003), (Makarova et al., 2015). In addition, *Drosophila* models have many notable achievements in age-related diseases, such as CVDs, sarcopenia and neurodegenerative diseases (Diop and Bodmer, 2015), (Hunt et al., 2021), (Feany and Bender, 2000), (Lu and Vogel, 2009).

Although exercise is an economical and effective treatment for age-related diseases (Li et al., 2020a), (Fiuza-Luces et al., 2018), (Batsis and Villareal, 2018), there are still many limitations in human and animal studies due to life cycle limitations (Blice-Baum et al., 2019). In recent years, *Drosophila* exercise models with short lifespan and mature genetic tools have become the optimal choice for researchers (Li et al., 2020b), (Kim et al., 2020), (Wen et al., 2016).

## 2 Development of a *Drosophila* exercise model for cardiovascular aging research

CVDs are the leading cause of death worldwide, with an estimated 17.9 million deaths from CVDs in 2019, accounting for 32% of global deaths (WHO (World Health Organization), 2017). Aging is a major risk factor for CVDs, including atherosclerosis, hypertension, myocardial infarction, and stroke (Paneni et al., 2017), (North and Sinclair, 2012). Exercise therapy is an economical and effective therapy to reduce mortality and risk of heart disease (Goenka and Lee, 2017). These studies are equally applicable in flies. In aged *Drosophila*, exercise enhances cardiac function and improves cardiomyocyte ultrastructure and heart failure (Piazza et al., 2009), (Li et al., 2020b). Nicotinamide adenine dinucleotide (NAD+) is a central metabolite associated with atherosclerosis, ischemic, diabetic, arrhythmogenic, hypertrophic or dilated cardiomyopathy, and different forms of heart failure (Abdellatif et al., 2021). Recently, several studies using *Drosophila* models have shown that NAD + supplementation improves mitochondrial mass, delays accelerated aging and extends lifespan through DCT-1 and ULK-1 (Fang et al., 2019). Consistently, mice prolong lifespan by supplementation with the NAD + precursor nicotinamide riboside (NR) (Zhang et al., 2016). In addition, high expression of NAD + synthase protein positively affected cardiac function in aging flies, including increased cardiac output and reduced heart failure (Wen et al., 2016). Similarly, NAD + precursor treatment also improved cardiac function in aged MDX mice with cardiomyopathy and improved mitochondrial and cardiac function in a mouse model of iron deficiency heart failure (Ryu et al., 2016), (Xu et al., 2015). During physical exercise, cellular energy requirements change all the time, including NAD + and NADH concentrations (White and Schenk, 2012). In mice, swimming increased NAD + levels in muscle (Cantó et al., 2010). In rats, endurance exercise resulted in a sustained increase in NAD + levels in the gastrocnemius muscle of young and aging rats (Koltai et al., 2010). A recent study showed that exercise increased cardiac NAD + levels and PGC-1α activity to improve lipotoxic cardiomyopathy in aged flies, which was associated with NAD+/dSIR2/PGC-1α pathway activation (Wen et al., 2019a). *Drosophila* dSir2, a homolog of mammalian Sir2, encodes deacetylase activity that prolongs flies lifespan (Rosenberg and Parkhurst, 2002), (Griswold et al., 2008). In addition, exercise activates the cardiac dSir2/Foxo/SOD and dSir2/Foxo/bmm pathways and reduces the occurrence of diastolic dysfunction as well as enhances cardiac contractility (Wen et al., 2019b). Exercise not only improves CVDs in aging *Drosophila*, but also resists the stress on the heart caused by a high-fat, high-sugar and high-salt diet. For example, lipid levels in the heart are significantly increased in dFatphet mutants, and exercise rescues myocardial lipid content and cardiac function (Sujkowski et al., 2012). Long-term exercise resists high salt-induced premature cardiac failure by blocking CG2196 (salt)/TOR/oxidative stress and activating dFOXO/PGC-1α (Wen et al., 2021a). Electrical pacing produced a significantly increased rate of heart failure when flies were exposed to a high sucrose diet (Bazzell et al., 2013). Aging is an important cause of arrhythmias (Chadda et al., 2018). In aged flies, early physical exercise improves arrhythmias, mainly by reducing the incidence of fibrillation and increasing the occurrence of bradycardia (Zheng et al., 2017). These facts show a great similarity, and humans compared to rats. For humans, exercise training is important for the prevention and treatment of CVDs (Lavie et al., 2015). For example, persistent regular exercise provides benefits in a variety of diseases, including atherosclerosis, atrial fibrillation, heart failure, and cardiac lipotoxic injury (Rognmo et al., 2012), (Risom et al., 2017), (Cattadori et al., 2018), (Schrauwen-Hinderling et al., 2010). In mice, the same beneficial effects of exercise on the heart have been reported (Fiuza-Luces et al., 2018), (Harris et al., 2020), (Vujic et al., 2018), (Börzsei et al., 2021), (Cheedipudi et al., 2020). In conclusion, this illustrates the significance of the *Drosophila* exercise model for the study of cardiac function.

## 3 Development of *Drosophila* exercise models in circadian rhythm studies

Aging leads to a weakening of circadian rhythms, such as the sleep/wake cycle. These rhythms are generated by biological clocks, which are based on cell-autonomous negative feedback loops involving clock genes that display molecular oscillations in approximately 24-h cycles. Clock genes are conserved from *Drosophila* to humans, and their oscillatory activity coordinates rhythms at the molecular, physiological and behavioral levels (Rakshit et al., 2013). *Drosophila* exhibits a sleep-like state that is regulated by both circadian rhythms and homeostasis (Shaw et al., 2000). It has been reported that knockdown of dATF-2 in pacemaker neurons decreases sleep duration, while ectopic expression of dATF-2 increases sleep duration (Shimizu et al., 2008). However, the degree of dATF-2 phosphorylation can be enhanced by forced exercise of the dp38 pathway (Shimizu et al., 2008). This suggests that dATF-2 is the regulator that links sleep to exercise. Furthermore, both chronic hypoxia and exercise improved sleep quality and climbing ability and extended maximum lifespan in aged flies, but exercise was insensitive to improvements in circadian rest/activity rhythms (Li et al., 2020b), (Zheng et al., 2017). Neuropeptide F (NPF) positive clock neurons have been reported to be critical for the control of nocturnal activity in *Drosophila* (Hermann et al., 2012). Interestingly, *Drosophila* exercise is similar to humans in maintaining and improving circadian rhythms (Rakshit et al., 2013), (Gabriel and Zierath, 2019). However, in *Drosophila*, the link between regular exercise and NPF remains poorly understood. Another study showed that exercise increased the duration of nighttime sleep by decreasing nocturnal activity, while also increasing the number of second deep sleeps and the intensity of daytime activity (Li et al., 2020b). Therefore, it will be interesting to study the effect of exercise and NPF on circadian rhythms. In contrast, one study reported that regular exercise did not improve circadian rhythms or lifespan in wild-type flies, but the clock mutant per 01 significantly reduced climbing ability with or without exercise, suggesting a role for some specific clock genes in maintaining health as part of healthy aging (Rakshit et al., 2013). The paradoxes that lead to the results may be due to differences in model building, including exercise devices, protocols and detection means. As an excellent model of circadian biology and aging, *Drosophila* is well suited to be combined with exercise to explore the molecular pathways between exercise and circadian rhythms. However, we must carefully consider the experimental errors caused by different exercise devices and protocols, otherwise revealing the intrinsic connection between exercise and circadian rhythms will become difficult.

## 4 Development of *Drosophila* exercise models in obesity-related diseases

Obesity is a global epidemic that is associated with aging and diet (Santos and Sinha, 2021). Obesity increases the risk of many health problems, including T2D, metabolic syndrome, CVDs, and cancer, and therefore leads to higher mortality (Aune et al., 2016), (Global BMI Mortality Collaboration Di Angelantonio et al., 2016). Physical exercise prevents obesity, reduces visceral fat and maintains body weight (Oppert et al., 2021), (Swift et al., 2018), (Villareal et al., 2017). *Drosophila* has become an excellent model for metabolic and diet-related diseases due to its powerful genetic tools and stable reproducible phenotype (Birse et al., 2010), (Musselman and Kühnlein, 2018). Although exercise is believed to mitigate the damage caused by obesity, it remains controversial (Waters et al., 2013). Therefore, the *Drosophila* exercise model serves as a bridge to reveal the intrinsic relation between exercise and obesity. *Drosophila* need only be fed a diet containing 30% coconut oil for 5 days to exhibit a phenotype similar to that of the mammalian metabolic syndrome, including increased glucose levels and decreased insulin-like peptide 2 (Dilp2) levels (Birse et al., 2010). The TOR pathway is associated with nutrient-sensing signaling in flies (Luong et al., 2006). Reducing the function of the TOR pathway may accelerate lipolytic metabolism and may also reduce lipid anabolism or storage (Birse et al., 2010). These results are similar to those in humans and rodents in that elevated TG levels induced by high-fat diets are associated with disruptions in lipid and glucose homeostasis, and mitochondrial function, which may lead to lipid accumulation and lipotoxic damage (Ouwens et al., 2005), (Unger, 2003), (Schaffer, 2003). Recent studies have found that regular exercise reduces aging-induced increases in cardiac triglycerides, which may be associated with activation of the cardiac dSir2 pathway (Wen et al., 2019b). Regular exercise is also able to increase antioxidant defense and control the production of RS required for cellular metabolic regulation, improving adiposity and glycemia (Dahleh et al., 2021). In addition, exercise may also reduce high-fat diet-induced whole-body hypertriglyceride levels by decreasing the expression of apoLpp (Ding et al., 2021). It is well known that high-fat diet-induced obesity induces cardiac lipid accumulation and leads to the development of lipotoxic cardiomyopathy. A study showed that lipotoxic cardiomyopathy can be reversed by exercise activation of the Nmnat/NAD+/SIR2 pathway (Wen et al., 2021b). These results have similarities with some studies in humans and mammals, such as exercise improving dyslipidemia and insulin resistance by reducing apolipoprotein B in patients with T2D (Alam et al., 2004), and in aged rats, exercise training promoting SIRT1 activity and improving antioxidant defenses in heart and adipose tissue (Ferrara et al., 2008). Another recent study showed that exercise and cold stimulation were able to alter the expression levels of the brown fat and beige fat markers ucp1, serca2b, β3-adrenergic receptor, prdm16, ampk, and camk, and reduce lipid accumulation (Huang et al., 2022). Although the above studies are not sufficient to prove whether exercise has an effect on lipid browning in flies, they provide indirect evidence, which suggests that exercise holds great potential in the regulation of lipid metabolism in flies.

## 5 *Drosophila* exercise model in skeletal muscle aging

In humans, the mortality and pathogenesis of many age-related diseases are related to the functional status, metabolic demands and mass of skeletal muscle, suggesting that skeletal muscle is a key regulator of whole-body aging (Anker et al., 1997) (Metter et al., 2002) (Nair, 2005) (Ruiz et al., 2008). In *Drosophila* melanogaster, the organization and metabolism of skeletal muscle fibers is similar to that of mammals (Piccirillo et al., 2014). But, muscles undergo more drastic age-related degeneration, which may be due to the lack of satellite stem cells and the limited muscle repair capacity of this organism (Grotewiel et al., 2005). A major difference between *Drosophila* and mammalian muscles is the lack of muscle stem cells. This feature makes *Drosophila* muscle, excellent models for identifying the mechanisms by which assembled sarcomeres are maintained and repaired without the confounding influence of regeneration as found in mammalian muscle (Christian and Benian, 2020). In addition, *Drosophila* muscle function can be analyzed by measuring their ability to fly and climb (Gargano et al., 2005). Due to these properties, flies are emerging as a useful model organism to study muscle aging together with mammalian models.

Skeletal muscle aging is a risk factor for the development of several age-related diseases, such as sarcopenia, metabolic syndrome, cancer, Alzheimer’s disease, and Parkinson’s disease (Christian and Benian, 2020), (Ruiz et al., 2011), (Demontis et al., 2013a). Exercise and muscle function are important predictors of age-related mortality in humans (Anker et al., 1997), (Metter et al., 2002), (Demontis et al., 2013b). For example, exercise protects transgenic mice with Alzheimer’s disease and Parkinson’s disease from neurodegeneration (Zigmond et al., 2009). Endurance exercise rescues mitochondrial defects and premature aging in mice defective in proofreading-exonuclease activity of mitochondrial DNA polymerase γ (Safdar et al., 2011). Another study showed that Sestrins are necessary and sufficient for beneficial adaptations to muscle function and metabolism in *Drosophila* and mice (Kim et al., 2020). Knockdown of Sestrins reduced endurance and flight in exercise-adapted flies (Sujkowski and Wessells, 2021). Similarly, knockdown of Sestrins in exercise mice impeded endurance and metabolic benefits (Sujkowski and Wessells, 2021). *Drosophila* muscle-specific dSesn expression replicates similar improvements in aging mobility by exercise and mediates changes in lysosomal activity in a variety of tissues, and both adaptations are dependent on TORC2-Akt activity and PGC1α (Sujkowski and Wessells, 2021). In addition, *Drosophila* muscle can play an important role in delaying aging (Rai et al., 2021). The first finding was that adult muscle-specific overexpression of dFOXO prolongs lifespan in *Drosophila* (Demontis and Perrimon, 2010). Although these findings underscore the fundamental role of muscle in regulating systemic aging, the molecular mechanisms involved in this inter-tissue communication are largely unknown.

Exercise not only produces beneficial effects in one’s own muscles but also has the potential to trigger beneficial effects in other tissues. Examples include increased energy expenditure and clearance of ectopic lipid stores (Hawley et al., 2014), improved insulin sensitivity and lower circulating insulin levels (Hawley et al., 2014), and increased secretion of exercise-regulated myocytokines, including irisin (Whitham et al., 2018) and extracellular vesicles (Arnold et al., 2011). Myokines can act on distant tissues such as adipose tissue, liver, pancreatic β-cells and endothelium (Pedersen and Febbraio, 2012). Insulin-like growth factor-1 (IGF-1) is an actin produced by muscles in response to exercise (Pedersen and Febbraio, 2012), (Hede et al., 2012). In *Drosophila*, ImpL2 is a member of the immunoglobulin superfamily, similar to mammalian IGFBP7, which binds to Dilps and inhibits insulin signaling and promotes mitochondrial autophagy (Owusu-Ansah et al., 2013). Mild muscle mitochondrial damage preserves mitochondrial function, inhibits age-dependent degeneration of muscle function and structure, and prolongs lifespan (Copeland et al., 2009), (Kirchman et al., 1999), (Dillin et al., 2002), (Liu et al., 2005). Although muscle-derived insulin-like growth factor binding protein is not detected in the circulation, it induces muscle hypertrophy after exercise in an autocrine/paracrine manner (Vinciguerra et al., 2010), (Silverman et al., 1995). It is well known that physical exercise counteracts the deleterious effects of secondary aging by preventing the decline in mitochondrial respiration, attenuating the loss of muscle mass associated with aging, and enhancing insulin sensitivity (Cartee et al., 2016). Although the *Drosophila* exercise model is not well studied in the field of skeletal muscle aging, its evolutionarily conserved myokines and short lifespan characteristics make it an excellent model for studying the role in intertissue communication.

## 6 Different genetic backgrounds of *Drosophila* exercise models

A growing number of studies have used *Drosophila* exercise to mimic phenotypes similar to those of humans, including increased endurance, improved age-related decreases in mobility and cardiac function, improved lipid metabolism, and increased lifespan (Wen et al., 2016), (Ding et al., 2021), (Lowman et al., 2018), (Piazza et al., 2009). *Drosophila* exercise is a complex multifactorial response and it has different exercise performance in different genetic backgrounds, including climbing speed and endurance (Damschroder et al., 2020). In addition, age, diet and gender are also factors that influence exercise performance. For example, climbing speed, endurance and flight performance decrease with age (Damschroder et al., 2020), (Sujkowski et al., 2019). The effect of diet on endurance is dramatic and affects acute endurance and adaptation to chronic exercise training, with diet composition having a greater effect than calorie content (Bazzell et al., 2013). In addition, the effect of gender on exercise is equally important; when looking at the distribution of activity levels during the same 2-h exercise session, there is a strong correlation between gender and exercise, with females experiencing an early burst of activity and males maintaining activity levels throughout the exercise session (Sujkowski et al., 2020). The mechanisms that lead to genotypic variation in exercise capacity are important to uncover the genetic pathways of exercise, and we should take advantage of cross-species genetics to better explore the interactions between exercise and aging-related diseases.

## 7 *Drosophila* exercise device

**FIGURE 1:**
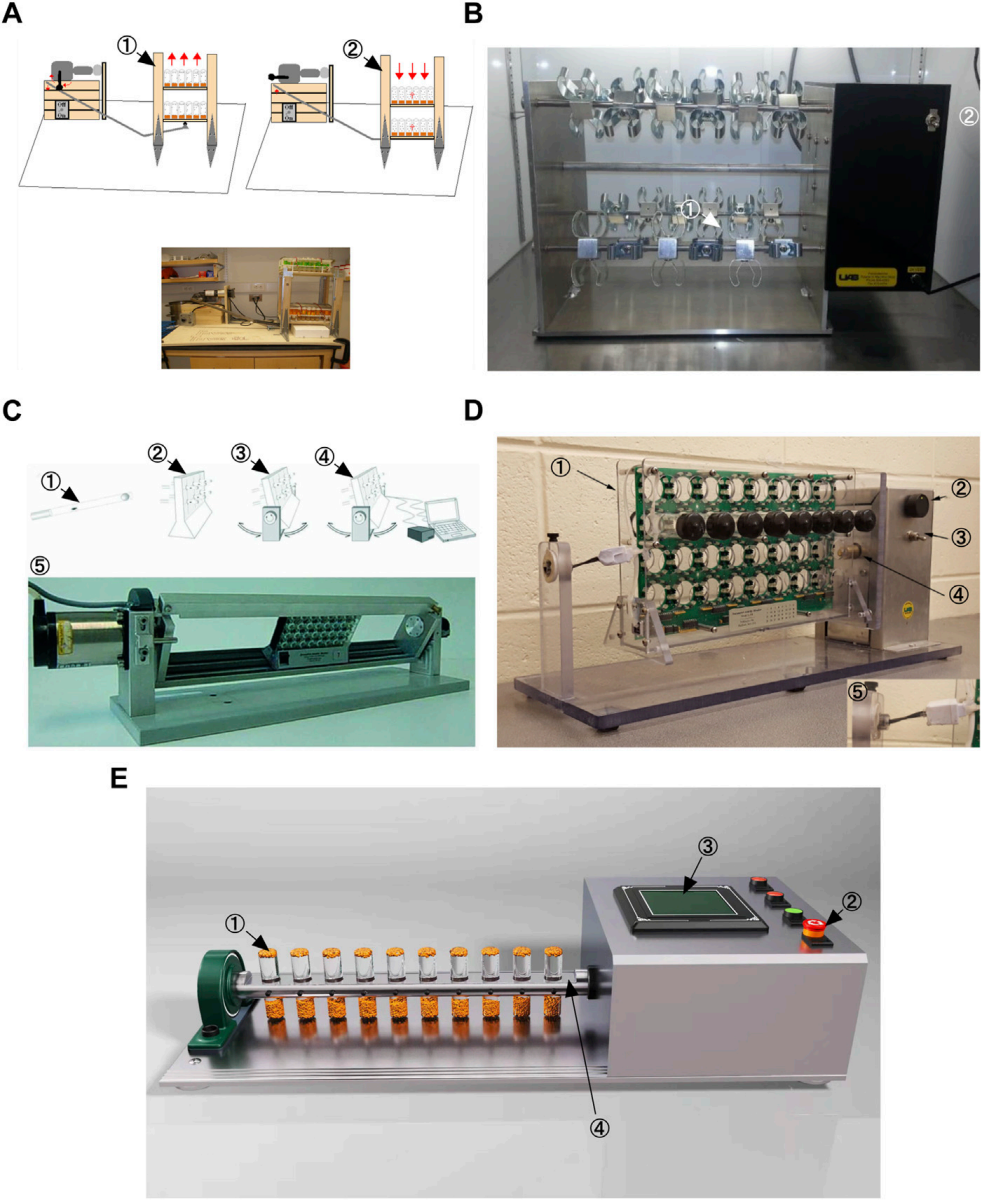
Different Drosophila exercise devices. **(A)** Power Tower. This is adapted from Piazza et al., 2009. **(B)** TreadWheel. This is adapted from Mendez et al., 2016. **(C)** Swing Boat. This is adapted from Berlandi et al., 2017. **(D)** REQS. This is adapted from Watanabe and Riddle, 2018 **(E)** Flip Bottle.

More than 40 years ago, scientists discovered that *Drosophila* exhibit an inherent behavior of crawling against gravity when at the bottom of a vial, a behavior commonly referred to as negative geotaxis (Miquel et al., 1976). Negative geotaxis in *Drosophila* requires Johnston’s organ, a mechanosensory structure located in the tentacles that also detects near-field sounds (Kamikouchi et al., 2009), (Sun et al., 2009). To date, five *Drosophila* exercise devices have been described. A decade ago, a first generation locomotion device, the Power Tower, was developed based on the negative tropism of *Drosophila* (Piazza et al., 2009). The Power Tower device lifts up flies fixed to a platform by a motor and then lets the platform fall freely with gravity, causing the flies to fall to the bottom of the bottle (Figure 1A). Due to their negative geostasis response, the flies crawl upward until the motor makes them fall to the bottom of the bottle once again. The TreadWheel device stimulates flies to crawl upward in a fully rotating manner and avoids some physical shocks during locomotion (Mendez et al., 2016), (Katzenberger et al., 2013) (Figure 1B). The Swing Boat device allows the tube to be rotated alternately 30° to each side and also allows the collection of data during locomotion in combination with the *Drosophila* Monitoring System (DAMSystem) (Berlandi et al., 2017) (Figure 1C). REQS is an upgraded version of the TreadWheel, similar to the Swing Boat, allowing the combination of a DAMSystem to quantify the level of movement of flies (Watanabe and Riddle, 2018) (Figure 1D). In addition, the Key Laboratory of Physical and Exercise Rehabilitation of Hunan Province also developed a *Drosophila* exercise device in an experiment in which a motor was controlled to drive the flip of the vial on the platform (Figure 1E). The difference is that each rotation of the device is 180 and this stimulates the flies to actively walk upwards inside the vial (Zheng et al., 2015). This device is called “Flip Bottle” because it keeps turning the bottle during its operation. All in all, the other four devices are mainly rotational in design compared to the Power Tower device. The “rotational” approach allows for greater avoidance of physical damage during the exercise of flies. Although the upgrade of the exercise device reduces the possibility of physical damage, there are still some questions about the efficiency of flies’ exercise in the vial and how to accurately determine the intensity of exercise. Various research protocols currently use motor rotation speed and time as key factors in determining exercise intensity (Katzenberger et al., 2013), (Berlandi et al., 2017), (Watanabe and Riddle, 2018). In short, Power Tower triggers *Drosophila* exercise through mechanical vibration, while the other four trigger exercise through rotation, including full rotation for TreadWheel and REQS, alternating 30 per side for Swing Boat, and alternating 180° per side for Flip Bottle. In addition, Swing Boat and REQS incorporate the DAMSystem, which makes them more objective in monitoring the intensity of exercise. However, in previous studies it was found that flies exhibited a passive tendency to climb after exercising for a period of time (usually after 20–30 min) (Watanabe and Riddle, 2017). This means that flies stay in a certain location in the vial and their range of movement decreases dramatically, which makes it more difficult to determine the intensity of exercise. It is well known that regular exercise can bring great benefits for healthy aging, and the emergence of *Drosophila* exercise models will further reveal the relationship between exercise and aging. Therefore, precise quantification of exercise intensity and the development of more advanced *Drosophila* exercise devices will become highly relevant in the future.

## 8 Summary

**FIGURE 2:**
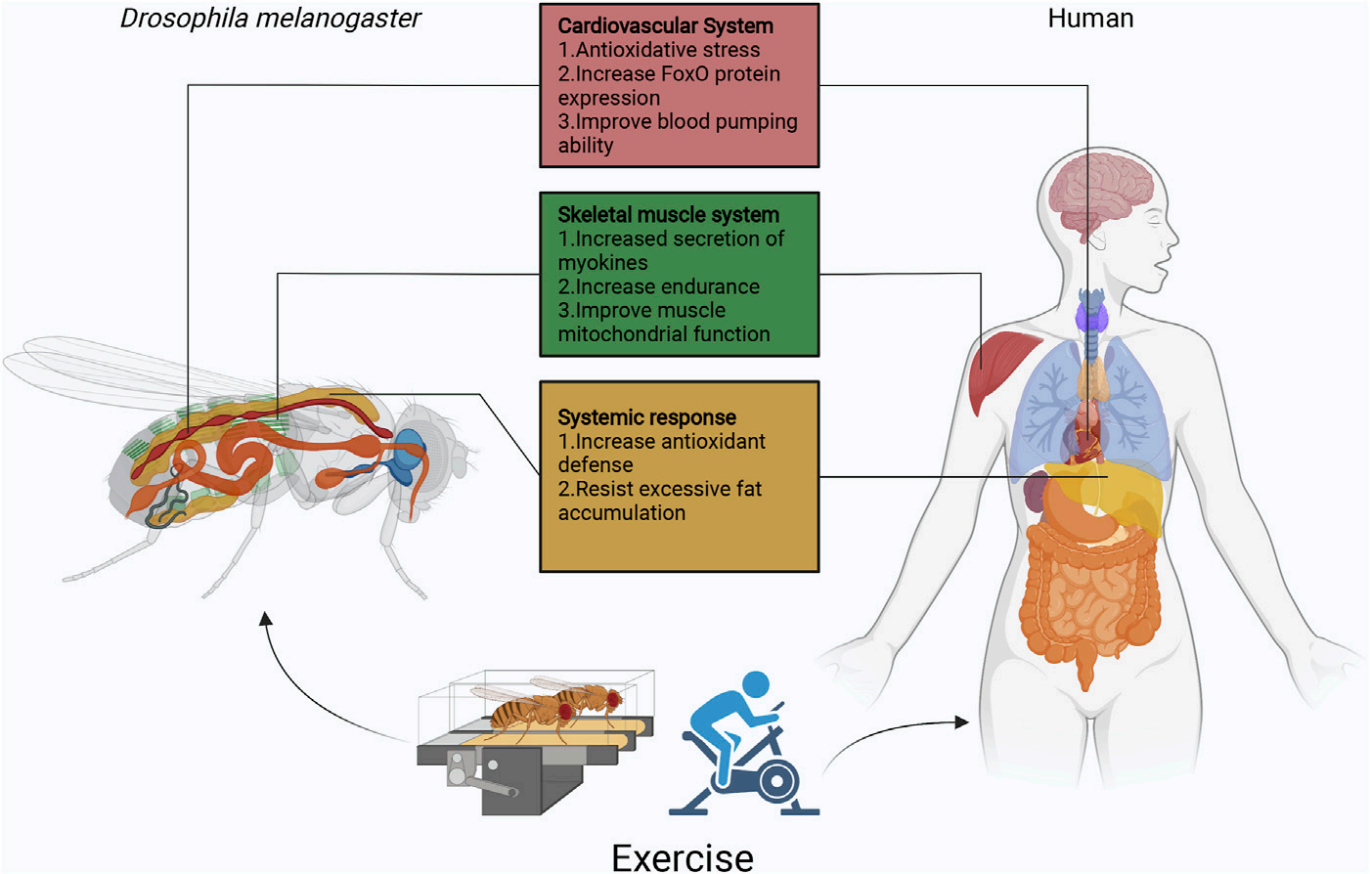
Overview of the function of *Drosophila* exercise in different organs. Currently, *Drosophila* exercise models have made some achievements in cardiovascular, skeletal muscle, and fat body. Exercise activates the cardiomyocyte dSir2/FoxO/SOD and dSir2/FoxO/bmm pathways to delay cardiac aging. In Sestrins, Sesn1 is mainly expressed in muscle, where it is involved in the metabolic response to exercise and is associated with TORC2-Akt activity and PGC1α. In addition, skeletal muscle secretes myokines (ImpL2) that inhibit insulin signaling and promote mitochondrial autophagy. Exercise though apoLpp to regulate abnormal lipid metabolism, it also activates Nmnat/NAD+/dSir2 to resist lipotoxicity. In exception to the heart, fat body and skeletal muscles, *Drosophila* has other systems similar to those of humans, such as the central nervous system represented by the *Drosophila* and human brain, the digestive system represented by the *Drosophila* and human intestine, the respiratory system represented by the *Drosophila* thorax and human lungs, and the reproductive system represented by the *Drosophila* and human ovaries/testes. The effects of exercise on aging individuals are complex, but the use of simple *Drosophila* exercise models will be exciting for exploring the role of exercise in different biological processes, while facing various difficulties and challenges.

*Drosophila* and other animal models are widely used to study the relationship between exercise and aging, such as mice and zebrafish (Murphy et al., 2021), (van Praag et al., 2005). But because of the complexity of these systems, we need simpler model organisms to overcome these challenges. *Drosophila* has the obvious advantage of being a first-line model for testing hundreds of potential longevity enhancers in multicellular organisms and can be easily adapted to mammalian models. *Drosophila* exercise produces physiological characteristics similar to those of humans (Figure 2). As described in this review, the *Drosophila* exercise model provides some new insights into cardiac aging, abnormal lipid metabolism, circadian rhythm disorders, and skeletal muscle aging. In addition to this, exercise regulates a variety of neurological disorders, including neuroendocrine, neurotransmitter, neuroinsulin signaling, antioxidant and anti-inflammatory responses, and cell survival and death pathways (Viru, 1992), (Meeusen and De Meirleir, 1995), (Lovatel et al., 2013), (Monteiro-Junior et al., 2015), (Kurgan et al., 2019), (Serra et al., 2019), (Kang et al., 2013). Surprisingly, however, *Drosophila* exercise models have only been reported in studies of octopamine and its receptors, a powerful neuromodulator that affects sensory and cognitive functions in insects (Zheng et al., 2015), (Sujkowski et al., 2017), (Farooqui, 2007). Many neuronal genes and neuronal transcriptional regulators were reported in a recent study to be affected by the exercise of the genus *Drosophila* (Watanabe and Riddle, 2021). Therefore, in the future, more studies will apply *Drosophila* exercise models to explore neurological disorders.

*Drosophila* exercise models have made remarkable achievements in this decade or so, but there are still some limitations awaiting the development of future methods and tools. Specifically, *Drosophila* exercise models are currently used primarily to study endurance exercise, but human exercise types also include exercises to improve strength, flexibility, and balance. In addition to increasing endurance, human exercise needs often include improving muscle strength or muscle tone, altering body composition, or increasing flexibility. Therefore, new methods and tools are needed to match *Drosophila* exercise to human exercise models. Although the *Drosophila* model has a limited redundancy of conserved pathways, it is still of great value for such studies. Therefore, it is important to further investigate the molecular mechanisms behind the physiological changes in exercise and aging. Furthermore, elucidating the role of exercise-related genes in *Drosophila* aging would provide evidence for a potential role of their human counterparts in aging (eg Table 1). *Drosophila* exercise models could provide therapeutic targets for exercise treatment of aging-related diseases. Of course these exercise-related genes need to be validated by extensive experiments before they hold promise as new therapeutic approaches.

**TABLE 1:** Exercise-related genes in *Drosophila* aging.

Gene	Participation path	Main findings	Reference
*spargel*		The PGC-1α *Drosophila* homolog spargel is required for adequate motor capacity in *Drosophila*	Tinkerhess et al. (2012)
*Sestrins*	TORC-1/TORC-2; AKT	Sestrins are necessary and sufficient for beneficial adaptations of muscle function and metabolism in *Drosophila* and mice	Kim et al., (2020), Sujkowski and Wessells, (2021)
*Pink1*		Pink1-expression of the mitochondrial proteome in *Drosophila* generally decreases in response to exercise	Ebanks et al. (2021)
*γ-oryzanol*		Combined use of γ-oryzanol and exercise enhances exercise capacity and viability in *Drosophila* without increasing cellular oxidative state	Kang et al. (2013)
*Nmnat*	NAD^+^/dSir2/FOXO	Cardiac Nmnat/NAD+/SIR2 pathway activation is an important underlying molecular mechanism by which endurance exercise and cardiac Nmnat overexpression protect *Drosophila* from lipotoxic cardiomyopathy	Grotewiel et al. (2005)
*salt*	salt/TOR/oxidative stress; dFOXO/PGC-1α	Endurance exercise improved the climbing capacity and survival in salt-overexpression *Drosophila*	Harris et al., (2020), Wen et al., (2020)
*ATXN2 Q117*		Endurance exercise has a significant positive effect on SCA2 (type of spinocerebellar ataxia) in *Drosophila*	Sujkowski et al. (2022)
*dSir2*	dSir2/Foxo/SOD; dSir2/Foxo/bmm	The activation of cardiac dSir2/Foxo/SOD and dSir2/Foxo/bmm pathways may be two important molecular mechanisms through which exercise works against heart aging in *Drosophila*	Cattadori et al. (2018)
*CG9940*	NAD (+)	Both normal expression and overexpression of CG9940 positively affected cardiac function, activity, and lifespan adaptation to exercise in aging *Drosophila*	Wen et al., (2016), Lavie et al., (2015)
*dFatp*		Endurance exercise can reverse increased lipid storage in the myocardium and deleterious cardiac function conferred by dFatp mutations	Schrauwen-Hinderling et al. (2010)
